# Inaugural Address

**Published:** 1911-01

**Authors:** Edgar G. Ballenger

**Affiliations:** Atlatna, Ga.


### INAUGURAL ADDRESS.

By Edgar G. Ballenger, M. D., Atlanta, Ga.

Mr. President and Gentlemen:

It is with feelings of hesitation and misgiving that I accept the honor and responsibility imposed by your election of me to the Presidency of the Fulton County Medical Society. Regarding the honor, I wish to express to you my deepest appreciation of it and also to hope that I may not prove entirely unworthy of your selection. Referring to the responsibility, I think there is more to be said. Every one must agree that we are not making of the society all that we should, our meetings are often poorly attended, the papers on the program are frequently not read, the clinical side of our work is greatly neglected, we have too few demonstrations of methods, specimens and research work, in fact, we lack the scientific enthusiasm which is the very soul of a medical society and which should dominate our meetings.

Let us consider for a moment the "cause and effect," related to some of these not flattering, but all too true, accusations. First, why is the attendance often small? Because the meetings are poor. Why are the meetings poor? Because the attendance is small. These two factors have cast a spell of indifference over us and have produced a condition which in medical language we call a "vicious circle." Can we not break this malign combination? I most assuredly think we can.

The honor of reading a paper before our association is carried too lightly by most of us; a greater premium should be placed upon the allottment of time in which to present original articles. The attendance, except when a sensational feature is anticipated, is so small that the essayist feels it hardly worth the expenditure of time, thought and work upon a paper to be read before almost empty chairs. On the other hand, there is no assurance that the essayists will be present with any papers at all and so rather than run the risk of wasting an evening upon an uncertainty, we go to the theatre, play bridge, read, study or do something that suits best our individual taste, and so stay away from the meeting. We must make our meetings so worth while that no one can afford to miss them. How can we improve the attendance and be assured that a program worth while listening to will be presented? By imposing a reasonable fine upon the members who do not present the papers for which they are scheduled, or provide a substitute. To use the program as a mere advertisement, to monopolize the time allotted for a paper and then not present it, is a gross discourtesy to the society as a whole and especially to the members who are interested in the subject expected and perhaps have inconvenienced themselves to be present. We are willing to pay our fines when we fail to attend the meetings of the hospital staff, so, why should not a similar rule be passed by our society to improve the meetings and thereby directly improve the attendance? It would not work a hardship upon any member, as usually but one or two papers are read during the year. No excuse should be acceptable, unless a paper is read by the essayist or a substitute, then a fine should be the penalty. Incidentally these fines might well be expended in providing cigars and beer for certain social meetings or informal "smokers" which would promote a closer feeling of comradeship among us. And so it is not illogical to reason that our program being more complete will attract a larger attendance and, finally, when the attendance is seen to be good, some of the essayists will probably work more in the preparation of their papers, for they will be ashamed to read a poor paper before a large crowd; while others will take advantage of the opportunity and privilege to bring before the society new methods, improvements, and research work, which, without a decent audience, might otherwise go unwritten or be less fully developed.

An excellent way to produce more scientific enthusiasm and inspiration would be to have occasional special meetings at which prominent men from large medical centers could present papers upon modern work in which they are especially interested. Such meetings should draw a good crowd and produce more interest in the association and thereby enhance its value. It would also tend to develop the perhaps smouldering scientific spirit, of which most of us have a certain amount, but which is often neglected on account of the needed impetus to start it into activity. Other socieies use this plan successfully and so can we. Such men as Murphy, of Chicago, Crile of Cleveland, Cabot of Boston, Barker of Baltimore, Deaver or Anders, of Philadelphia, Janeway or Coley, of New York, and many others of like eminence would be honored to be present at special meetings for them. It is the custom, in extending invitations to address such meetings for the society also to offer to defray the actual necessary traveling and hotel expenses. I am sure many of our members would gladly take into their homes the guests of these occasions and give them more comfortable accommodations than could be obtained in hotels. Most men do not wish to have their expenses paid by the society and consequently the cost of a meeting of this kind would be very small compared with the increased interest that would be aroused and with the new members who would come into the society because of its greater value and usfulness.

The clinical side of our society work is greatly neglected. The probable cause of this is that "What is everybody's business is nobody's business." Is there not some one present who would hold himself responsible for arranging that the demonstration of at least one patient or specimen be given at every meeting? In this capacity he would head, assist and direct the work of the Case Committee. Many useful and instructive facts might be brought out if we could promote more interest in this phase of our duties and possibilities. Our work should be of such quality that the knowledge gained at each meeting would help every physician in attendance to treat his patients more successfully and more scientifically. This would make us more interested in our work, for we all need interest, ENTHUSIASTIC INTEREST that impresses the patient with our conscientious endeavor and removes from our work its drudgery.

There are many matters of importance which could be dwelt upon if time did not forbid. Especially is this true of the subjects which are within the province of the committees on Public Education, and Public Health and Legislation. Commendation is due the members who have recently arranged certain meetings to which the women's clubs have been invited. The large number present and eagerness with which information was sought concerning the important subjects discussed was all out of proportion to the meager attendance of our own members. It is such matters as public health, hygiene, and the prevention of disease, rather than its cure, that we can show to the world in a convincing manner our superiority to the sectarians with their one-sided, limited curative methods.

Will you not lend us your support during the coming year? Without it our best efforts will be futile. Please do not stay away because you think our meetings are not worth while. Your presence in itself will ultimately help to improve the QUALITY of the papers and thus MAKE the meetings better.

